# The development and user evaluation of health behaviour change resources for teenage and young adult Cancer survivors

**DOI:** 10.1186/s40900-019-0142-6

**Published:** 2019-02-15

**Authors:** Gemma Pugh, R. Hough, H. Gravestock, C. Davies, R. Horder, A. Fisher

**Affiliations:** 1 Department of Behavioural Science & Health, University College London, 1-19 Torrington Place, London, WC1E 6BT UK; 2 Centre for Sports & Exercise Medicine, Queen Mary University of London, London, UK; 3 Department of Haematology, University College London Hospital, London, UK; 4 CLIC Sargent, No. 1 Farriers Yard, Assembly London, 77-85 Fulham Palace Road, London, UK

**Keywords:** Cancer, Adolescent, Young adult, Health behaviour intervention

## Abstract

**Plain English summary:**

This paper describes the methods that were used to develop a health behaviour intervention specifically for teenage and young adult cancer survivors (TYACS). The program of work, carried out in partnership with CLIC Sargent (a UK based cancer charity for children and young people) was guided by The Behaviour Change Intervention Design Process. A systematic review of existing intervention studies was carried out and TYACS were surveyed on their interest in receiving health behaviour information and their preference regarding the format, delivery and timing of such information. Health professionals were also surveyed to gather their views on how health behaviour information would be best delivered to young people with cancer. The results of these studies informed the development of a collection of health behaviour change intervention resources containing comprehensive lifestyle information and behaviour change support tools. TYACS and TYA health professionals were invited to review and provide feedback on the relevance, appeal and usability of the resources. It is hoped that by involving TYACS and TYA representatives at every stage of intervention development the problem of low uptake and adherence commonly encountered during intervention piloting will be prevented.

**Abstract:**

**Background**

Teenage and Young Adult Cancer Survivors (TYACS) are advised to adopt a healthy lifestyle in order to reduce the impact of cancer and its treatments upon their long-term health. However, at present there are no interventions available in the UK to support TYACS to lead a healthy lifestyle. To inform the development of a lifestyle intervention for TYACS a partnership was set up between academic behavioural scientists and CLIC Sargent, a cancer charity which supports children and young people.

**Methods**

A series of studies to understand patient and professionals needs and perspectives regarding health behaviour change were carried out. TYACS were surveyed to gather data on their current health behaviour status; interest in, and experience of receiving, lifestyle advice; and preference regarding the type, format, and delivery of a lifestyle intervention. Health care professionals were surveyed simultaneously to gather their views on how best to promote health behaviour change to TYACS. In this paper we summarise key findings from the development work, the resulting lifestyle intervention, and new data from a preliminary evaluation study exploring TYACS and TYA health professionals’ views on the relevance, appeal and usability of the intervention resources.

**Results**

A collection of health behaviour change intervention resources containing lifestyle information and behaviour change support tools were developed. These intervention resources were well received by TYACS and health professionals with the majority rating the information as high quality, helpful and relevant. Over 80% of TYACS reported they would find the support tools ‘very appealing’ or ‘quite appealing’. TYACS and health professionals provided feedback on how the resources could be improved including commenting that more personalized or tailored health behaviour information would be beneficial.

**Discussion and conclusion**

It is hoped that by involving TYACS and TYA representatives at every stage of intervention development,and carrying out a preliminary evaluation of the intervention resources, the problem of low uptake and adherence commonly encountered during formal intervention piloting and evaluation will be prevented.

**Electronic supplementary material:**

The online version of this article (10.1186/s40900-019-0142-6) contains supplementary material, which is available to authorized users.

## Background

There are currently over 2 million cancer survivors living in the UK; of these approximately 17,000 individuals are TYACS aged between 13 and 24 years [1]. Although this is proportionally low and accounts for less than 2% of the total number of cancer survivors, the majority of young people who have had a cancer diagnosis will suffer a psychosocial or physical health problem as a result of their cancer diagnosis [2–5]. These health difficulties often arise during treatment and persist throughout the lifecourse. Research now must focus on ways of improving the long-term health and well-being of young people with cancer [2].

Among adult cancer survivors there is trial and observational evidence linking positive health behaviours (physical activity, good nutrient and dietary intake, smoking abstinence, low levels of alcohol consumption, and skin protection) to better health outcomes [6–8]. There is some emerging evidence among TYACS that the same holds true and that supporting and encouraging TYACS to lead a healthy lifestyle may help reduce the impact cancer has upon both their current and long term health [9]. However, while several interventions designed specifically for TYACS have shown promising results, most were piloted within small feasibility studies and none were conducted in a UK health-care setting [10, 11]. Moreover, many of these existing health behaviour interventions were resource intensive, had high levels of attrition and did not provide sufficient long-term follow-up of health behaviour change. Such as: acceptability of the intervention to the target group; acceptability of the intervention to stakeholders and delivery organisations; if the content of the intervention is satisfactory to meet the needs of the target population; and if intervention format and delivery methods are appropriate [12].

The aim of this programme of work was to develop a health behaviour intervention specifically for TYACS. In this paper we summarise key findings from the development work, the resulting lifestyle intervention, and new data from a preliminary evaluation study exploring TYACS and TYA health professionals’ views on the relevance, appeal and usability of the intervention resources. Specifically within the preliminary evaluation study we aimed to determine i) if the health behaviour information developed as part of the proposed intervention meets the needs of TYACS and TYA health professionals and ii) if TYACS and TYA health professionals are receptive to the inclusion of discrete behaviour change techniques within the resources. It is hoped that by involving TYACS and representatives at every stage of intervention development the problem of low uptake and adherence commonly encountered during intervention piloting and evaluation will be prevented.

### Part 1. Developing the health behaviour intervention for TYA Cancer survivors

Several frameworks have been published to assist researchers and practitioners to develop interventions in a logical evidence based way [13]. The development of the health behaviour intervention for TYACS was guided by the Behaviour Change Intervention Design Process [14]. To ensure an intervention has maximal uptake and impact, this approach to behaviour change intervention design encourages identifying and understanding key behavioural issues and identifying how the behaviour change intervention should be delivered. Within this programme of work a systematic review of existing intervention studies was carried out to identify common characteristics of successful interventions [10]. Data on TYACS current health behaviour was then gathered to define and understand the need for a health behavior change intervention designed specifically for young people with cancer. Data on TYACS past experience of receiving lifestyle information and preference regarding health behavior information was also collected to identify intervention functions and options for delivery and implementation [15, 16]. Data on health professionals views of delivery of health behavior change to TYACS were also gathered [17].

### Collaboration with CLIC Sargent

This program of work was funded in part by an IMPACT studentship supported by CLIC Sargent and University College London. CLIC Sargent are a charitable organization in the United Kingdom who provide financial, practical and emotional support to children and young people affected by cancer. The policy and research team at CLIC Sargent provided initial advice on how best to design the content of the studies included within this project. The protocol and methodology of the studies were presented to CLIC Sargents Young Peoples Reference Group (YPRG) for feedback before the commencement of any studies. Following the advice of the YPRG the visual design of the health and lifestyle questionnaire reflected the visual identity of CLIC Sargent and included the charities logo upon the front cover. Young people within the group felt that it was important that the research materials were recognizable as being CLIC Sargent affiliated. The participation team at CLIC Sargent were instrumental in assisting with recruitment; all studies within this programme of work were advertised through the charities participation network and social media pages. The participation team also sent multiple reminders over the course of the study period. The web link to the health professionals survey was cascaded through the mailing lists of services staff employed or affiliated with CLIC Sargent. Following the intervention development studies the proposed intervention resources were written and developed in alignment with CLIC Sargents Information Standard™ accreditation. The final versions of the health behaviour information and resources were reviewed by CLIC Sargents information team who provided feedback on the content, language and layout of the intervention materials. CLIC Sargents were instrumental in ensuring the intervention resources were developed to reflect the needs of young people with cancer.

### Input and feedback from TYA representatives

Feedback from academics, health professionals and young people with cancer was received from the following groups: London Cancer Network, North Thames TYA Cancer Coordinating Group, CLIC Sargents’ YPRG, National Cancer Research Institute (NCRI) Psychosocial Oncology & Survivorship Clinical Studies Group (CSG), NCRI Teenage and Young Adult CSG, and Surrey University, Cancer Care Research Group. Individual comments from the members of these groups contributed to the planning and methodology of the studies which guided the development of the intervention resources.

### The health behaviour change intervention resources

The full details of the studies conducted to inform the development of the intervention materials have been published elsewhere [15–17]. In brief, TYACS demonstrated a preference for health behavior information available in multiple formats which accounts for the individual needs of young people with cancer. Social support and self-efficacy were found to be facilitators of behaviour change among TYACS, with many young people reporting that they were interested in resources which could support them to break unhealthy habits. These findings were reflected by TYA health professionals who reported lack of information was one of the main barriers to providing TYACS with information and support about lifestyle behaviour.

The results of these studies informed the development of a collection of health behaviour change intervention materials. The resources consisted of health behaviour information and behaviour change support tools. The intervention materials were based on habit theory and designed to prompt TYACS to make healthy lifestyle choices habitual [18]. The health behaviour information contained reference to the importance and benefits of healthy lifestyle behaviours, how to make sustainable lifestyle behaviour changes, and information on the health consequences of not meeting current Childrens’ Oncology Group recommendations. Specifically, the information materials highlighted TYACS increased risk of chronic disease and secondary primary neoplasms. This is in line with the long-term follow up guidance set by the Children’s Oncology Group which states, TYACS (particularly those with metabolic syndrome or cardiovascular disease) should be provided with comprehensive and detailed information on the importance of health behaviours and be fully informed of the benefits of physical activity and healthy dietary behaviours and the risks of smoking, heavy alcohol consumption and sun exposure. Based on findings from the intervention development work it was important that the information developed addressed issues specific to the needs of TYACS such as the impact of physical side-effects of cancer treatment, changes in taste preferences, and limitations in physical activity capability. Similarly, young people within the qualitative study talked about the importance of knowing how other TYACS had overcome these barriers to leading a healthy lifestyle. Data from research exploring how best to communicate cancer prevention among the general adolescent population suggest health promotion programmes and interventions not only focus on the ‘*Dos*’ and ‘*Don’ts*’ of leading a healthy lifestyle but challenge how young people to process the benefits and risks of certain health behaviours using case scenarios [19, 20]. Examples of TYACS dealing with a variety of barriers to leading a healthy lifestyle were therefore included within the resources. These case scenarios were designed to be self-reflective educative tools to empower young people to be more aware of their beliefs and behaviours.

The intervention resources contain behaviour change techniques disguised as ‘support tools’ which are designed to prompt young people to change their behaviour**.** Table 1 outlines the behaviour change techniques selected for inclusion within the behaviour change intervention resources. The illustrations of the goal setting, action planning, prompts/reminders, rewards, self-monitoring and social support health behaviour change ‘support tools’ developed during this programme of work are contained within Additional file 1. It was important that the behaviour change techniques included could be used flexibly and adapted to young people with differing needs and behaviour change goals. ‘Healthy Life Action Plan’ sheets were included within the resources and were designed to encourage TYACS to set goals to change their desired behaviour, to plan strategies to fit their new behaviour goal into their daily routine, and track their progress towards their desired goal overtime. The resources also included prompts encouraging TYACS to self-reward themselves for progress made towards their desired behavioural goal and space for TYACS to brain-storm ideas to overcome barriers to behaviour change.

**Table 1:** Content and rationale behind behaviour change techniques selected for inclusion within the intervention

Behaviour Change Technique (BCT)	BCT Definition [30]	Intervention Feature
Goal Setting (behaviour & outcome)	Set or agree a goal defined in terms of the behaviour to be achieved and the positive outcomes of the behaviour change.	The paper based, online and app based intervention materials will encourage TYA cancer survivors to identify a target behaviour and a set goals to change.
Problem Solving	Analyse, or prompt the person to analyse, factors influencing the behaviour and generate or select strategies that include overcoming barriers and/or increasing facilitators.	The paper based, online and app based intervention materials encourage TYA cancer survivors to think creatively of strategies to change their behaviour by writing down ideas which might work for them.
Action Planning	Prompt detailed planning of performance of the behaviour (must include at least one of context, frequency, duration and intensity).	The paper based, online and app based intervention materials will contain ‘Healthy Life Action Plan’ sheets which are designed to encourage participants to plan how they will fit the new action into their existing routines.
Behaviour Substitution	Prompt substitution of the unwanted behaviour with a wanted or neutral behaviour.	The paper based online and app based intervention materials contain information and tips ‘ideas for everyday change’ on new actions which could be incorporated into a daily routine to help TYA cancer survivors meet their daily goals.
Habit Formation	Prompt rehearsal and repetition of the behaviour in the same context repeatedly so that the context elicits the behaviour.	The paper based online and app based intervention materials will contain advice about forming habits and specifically repeating the same actions in similar contexts.
Habit Reversal/Substitution	Prompt rehearsal and repetition of an alternative behaviour to replace an unwanted habitual behaviour.	The paper based online and app based intervention materials will contain information on the importance of repeating new actions.
Feedback on behaviour	Monitor and provide information or evaluative feedback on performance of the behaviour.	The online and app based interventions will contain features where young people receive a score or result based upon their health behaviour.
Self-monitoring of behaviour	Establish a method for the person to monitor and record their behaviour as part of a behaviour change strategy.	The paper based, online and app based intervention materials will contain ‘Healthy Life Action Plan’ sheets which are designed to encourage participants to monitor the change in their behaviour over time.
Information about health consequences	Provide information about health consequences of performing the behaviour.	The paper based online and app based intervention materials will contain information about the potential benefits and costs of either having a healthy lifestyle or not having a healthy lifestyle.
Prompts/ Cues	Introduce or define environmental or social stimulus with the purpose of prompting or cueing the behaviour. The prompt or cue would normally occur at the time or place of the performance.	The intervention app will contain a feature to send automated reminders to TYA cancer survivors phones prompting healthy lifestyle behaviours.
Social Support (un-specified)	Advise on, arrange or provide social support (e.g. from friends, relatives, colleagues, buddies and staff) or non-contingent praise or reward for performance of the behaviour. It includes encouragement and counselling, but only when it is directed at the behaviour.	The paper based, online and app based intervention materials will advise participants to find a close friend or family member to encourage continuation with the behaviour change programme. The online intervention will include a message forum encouraging young people to communicate and praise one another for their achievements. The message forum will also encourage young people to share how they have changed their behaviours, the barriers they came across and how they addressed these challenges.
Social Comparison	Draw attentions to others’ performance to allow comparison with the persons’ own performance	The online intervention will include a message forum where TYA cancer survivors will be able to share and compare their behaviours with other young people.

In the past, leaflets and websites have been successfully used as platforms for intervention delivery among TYACS. However within the development work young people and health professionals stressed the importance of lifestyle behaviour available in multiple formats to suit individual needs. Illustrations of the resources in the form of a website online, an app or a short leaflet were developed. The proposed online and app based intervention resources contained features to allow young people to support one another and share information on health behaviour change. The proposed app also contained a function for TYACS to receive prompts to sustain positive health behaviour changes.

### Part 2. The preliminary user evaluation

The written health behaviour information and illustrations of the health behaviour change support tools were incorporated into an online survey evaluation survey. The online survey comprised of two parts. In Part 1 participants (both TYACS and health professionals) were asked to review and evaluate the written information for standard, quality and relevance. Within Part 2 of the evaluation survey, participants were asked to review and evaluate the illustrations of the proposed behaviour change support tools for appeal and usability. Two versions of the survey were created; one for young people with cancer and one for health professionals.

A total of 18 TYACS (mean age: 20 ± 3.12 years, 65% female) and 19 TYA health professionals (62% nurses, 19% social workers, and 5% physicians) took part in the online survey. Most TYACS had completed active treatment (83.4%) and reported a diagnosis of either leukaemia or lymphoma (55.5%). TYA health professionals predominantly worked in principal treatment centres with TYA patients with varying types of cancer. Most professionals (81%) had worked with TYA cancer patients for more than 2 years.

**Fig. 1:**
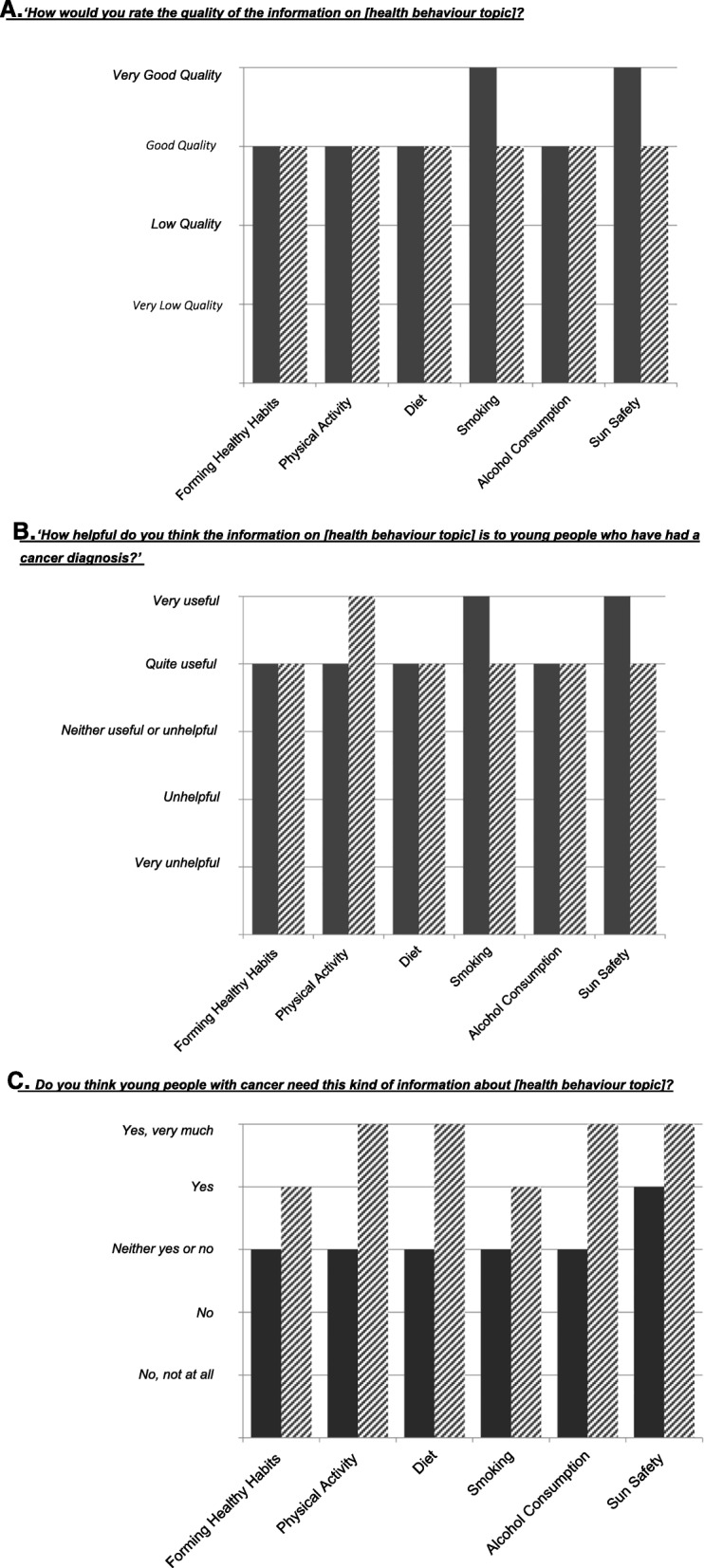
Quality, utility and relevance ratings of the TYA health behaviour change information

TYACS and health professionals views on the quality, utility and relevance of the information on each health behaviour topic are displayed in Fig. 1a**,** b and c respectively. Very few TYACS or health professionals regarded the information as low quality, unhelpful or irrelevant with most reporting that the information was of either *‘very good’* or *‘good’* quality and *‘very useful’* or *‘quite useful’* to young people with cancer. Specifically, the majority (54.5–72.7%) of young people thought the information on smoking and sun safety was of ‘*very good’* quality and *‘very useful’* to young people with cancer. Most TYA health professionals held the view that young people with cancer *‘very much’* needed this kind of health behaviour information whereas TYACS reported being more unsure if the information was needed selecting *‘neither yes or no’.* No health professionals or TYACS thought the information was irrelevant.

Table 2 displays participants’ views on the standard of the written information. All of the TYACS surveyed reported that the information was clear and that the language and tone used was appropriate for young people with cancer. However, only 36.4% (*n* = 4) of TYACS and 29.4% (*n* = 5) of health professionals felt the information was engaging for young people. Feedback on specific aspects of the information was positive with more than 80% of TYACS and over 60% of health professionals indicating they ‘*liked*’ or ‘*loved*’ the information on the benefits of a healthy lifestyle, information on the risks of an unhealthy lifestyle or the ‘ideas for everyday change’ sections.

**Table 2:** TYA cancer survivors and TYA health professionals’ views on the standard of the health behaviour information

	TYA Cancer Survivors % (n) (*n* = 11)*			TYA Health Professionals % (n) (*n* = 17)*		
	Yes	Partly	No	Yes	Partly	No
*Is it clear what the information is about?*	100 (11)	0 (0)	0 (0)	82.4 (14)	17.6 (3)	0 (0)
*Do you understand the language used?*	100 (11)	0 (0)	0 (0)	70.6 (12)	29.4 (5)	0 (0)
*Is the information written at the right level?*	100 (11)	0 (0)	0 (0)	64.7 (11)	23.8 (5)	4.8 (1)
*Does the information engage you as a reader?*	36.4 (4)	63.6 (7)	0 (0)	29.4 (5)	64.7 (11)	5.9 (1)
*Is the information written in the right tone?*	81.8 (9)	11.1 (2)	0 (0)	52.9 (9)	47.1 (8)	0 (0)

*Missing data due to survey drop-out: TYA cancer survivors (*n* = 7); TYA health professionals (*n* = 2

As displayed in Table 3 the majority (> 80%) of TYACS and health professionals viewed the behaviour change support tools as ‘*very appealing’* or *‘quite appealing’.* Only one TYACS reported that they did not find any of the behaviour change support tools interesting. If these health behaviour change resources were available today, 92.9% (*n* = 13) of health professionals reported that they would be extremely likely to signpost young people towards the resources. Sixty percent of TYACS said they would be either *‘extremely likely’* or ‘*very likely’* to use the resources and 80% (*n* = 4) said they would definitely recommend the resources to other young people with cancer.

**Table 3:** TYA cancer survivors and TYA health professionals’ views on the appeal of discreet behaviour change techniques

	TYA Cancer Survivors (*n* = 6)	TYA Health Professionals (*n* = 15)
Goal Setting		
Very Appealing/ Quite Appealing	85.7 (6)	66.7 (10)
Not appealing/ Not at all appealing	14.3 (1)	33.3 (5)
Action Planning		
Very Appealing/ Quite Appealing	83.3 (5)	73.3 (11)
Not appealing/ Not at all appealing	16.7 (1)	26.7 (4)
Prompts/Reminders		
Very Appealing/ Quite Appealing	83.3 (5)	86.7 (13)
Not appealing/ Not at all appealing	16.7 (1)	13.3 (2)
Self-Monitoring		
Very Appealing/ Quite Appealing	83.3 (5)	85.7 (12)
Not appealing/ Not at all appealing	16.7 (1)	14.3 (2)
Rewards		
Very Appealing/ Quite Appealing	83.3 (5)	93.3 (14)
Not appealing/ Not at all appealing	16.7 (1)	6.7 (1)
Social Support		
Very Appealing/ Quite Appealing	83.3 (5)	80 (12)
Not appealing/ Not at all appealing	16.7 (1)	20 (3)
Social Comparison/ Modelling		
Very Appealing/ Quite Appealing	83.3 (5)	100 (15)
Not appealing/ Not at all appealing	16.7 (1)	0 (0)

*Missing data due to survey drop-out: TYA cancer survivors (*n* = 7); TYA health professionals (*n* = 4)

TYACS reported that they would be most likely to use the resources if available as paper leaflets or booklets (*n* = 3, 60%) or as an app (*n* = 2, 40%). Health professionals on the other hand reported they were most likely to signpost young people towards a mobile app (*n* = 9, 64.3%) containing the resources. No health professional said they would signpost a young person in their care towards paper based resources. Most young people felt the resources would be best introduced during (20%) or immediately after treatment (40%) whereas most health professionals (*n* = 6, 43%) felt the resources should be delivered 3–5 months after treatment.

### Discussion - delivering a best practice intervention to TYACS

Often behaviour change interventions are developed with minimal understanding of the behavioural needs of the target population and minimal understanding of what functions the intervention should contain and how the intervention should be delivered [13]. This programme of work involving TYACS and TYA health professionals provided a thorough insight into these issues and resulted in a behaviour change intervention which reflects the needs of young people with cancer. It is hoped that by developing the health behaviour change intervention resources to be reflective of the needs and preferences of young people, the ensuing intervention is more likely to be effective at prompting sustainable behaviour change [21].The formative evaluation of the intervention materials provides important insight into TYACS and TYA health professionals’ receptivity to the intervention resources.

Findings from this program of work indicate that the written health behaviour change information was generally well received with the majority of TYACS and TYA health professionals rating the information as high quality, helpful and relevant. Feedback on the written standard of the information was also high with 100% of TYACS reporting the information was clear, understandable and written at the right level. Over 80% of young people *‘*liked’ or ‘loved’ information on the benefits of a healthy lifestyle; information on the risks of an unhealthy lifestyle; the ‘ideas for everyday change’ and the information about forming new habits. Health professionals were more critical of the information contents than TYACS with some raising questions about the suitability of providing general health behaviour advice to young people with cancer especially if the young person in question has very specific care needs. Nevertheless, in comparison to TYACS, a greater proportion of health professionals viewed the information as relevant and needed. TYACS may be apathetic towards their needs for health information. Apathy, defined as a lack of motivation relative to ones level of functioning [22], manifests in the form of diminished goal-directed cognition and behaviour (e.g. lack of effort to do things and lack of interest in new experiences) and diminished emotional responsivity to both positive and negative events. Apathy often co-occurs alongside neurological disorders such as depression, schizophrenia and Alzheimer’s disease. The literature regarding apathy and health among adolescents is scant however, one study of adult survivors of childhood posterior fossa brain tumours (*n* = 117, Mean age 32 years: Age range: 18–53) found 35% of survivors reached or exceed the Marin Apathy Evaluation Scale score for diagnosis of apathy [23]. Psycho-educational interventions which stimulate care and interest in health behaviour among TYACS may increase young peoples’ engagement with health behaviour information and behaviour change resources.

Many TYACS and health professionals commented that more specific personalized or tailored health behaviour information would be beneficial. The intervention materials in the current format are ‘targeted’ towards TYACS in that they provide a specific message to a specific population [24]. Future work should explore how the information and behaviour change support components of the intervention could be made personal to the user. In the past, receiving personalized feedback on health behaviour data entered into the intervention has previously been used as an effective means of providing tailored personal health behaviour change support to TYACS [25]. Other methods may include allowing TYACS to pick behaviour change modules or information content that they deem most relevant to them. Within one review of digital health interventions for children and young people with mental health problems (depression, anxiety, eating disorders, attention deficit hyperactivity disorder (ADHD), and psychosis) it was suggested that the ability of young people to personalize even small features (e.g. background colour, gender or the appearance of the homepage) influences how users engage with the intervention [26]. Further research is required to explore if health behaviour change interventions for TYACS should be personalized by the individual, by cancer type, or by age and determine how this may influence intervention uptake and engagement.

TYACS and health professionals’ feedback on the behaviour change support tools was also positive. Over 80% of young people who were surveyed reported that they would find the support tools ‘very appealing’ or ‘quite appealing’. Health professionals held similar views and thought rewards and social comparison/ modelling would be most appealing to TYACS. These findings strengthen the hypothesis that behaviour change resources designed specifically for TYACS might have a widespread use. Moreover, the finding that young people with cancer find such behaviour change techniques appealing is consistent with previous research conducted among the general population. For example, within one study exploring the user requirements of a lifestyle behaviour change app, young people (*n* = 16, Mean age: 24 years) reported that such an app designed to promote healthy lifestyle choices should include four main features: behaviour tracking, health goal setting, reminders and tailored information [27]. Participants within the study by Ribeiro and colleagues implied that the behaviour tracking element was an important feature as it was likely to be a ‘call to action’ for behaviour change. This is consistent with evidence which indicates experiential feedback is a key component of successful behaviour change interventions [28]. Additional research is needed to determine the extent to which TYACS would voluntarily engage with such behaviour change techniques embedded within the intervention resources.

Positive feedback from both young people and health professionals on the content and delivery of the resources provide assurance that these behaviour change intervention materials will be useful to young people with cancer. However it is important not to place too much emphasis on the positive feedback from TYACS and health professionals as there is a possibility of social desirability bias. The information sheets outlining the study included reference to the fact that researchers from the Department of Behavioural Science and Health at UCL developed the information materials and behaviour change support tools in partnership with CLIC Sargent; therefore it is possible that young people and health professionals were less likely to criticise the intervention resources as they may have known it would be researchers from UCL looking at their feedback. Similarly, participants may have noted CLIC Sargent’s input and assumed the information materials to be credible and reliable and therefore not been as critical as perhaps they could have been. It is also unlikely that the views of young people and health professionals within this study reflect the views of the entire TYACS population as levels of health literacy and age may affect a young persons’ ability to comprehend or engage with the information and materials evaluated within this study. A number of TYACS also experienced a high-level of survey fatigue and dropped out of the survey after Part 1. This is unsurprising as reading and reviewing the resources was a time consuming task. Future work should therefore explore TYACS’ thoughts on behaviour change support tools in a larger sample. Future work should also include ‘think-aloud’ studies to understand how young people of different ages and cancer types may view and engage with the intervention resources, which parts of the intervention could be made redundant, and how the intervention could be made more persuasive to behaviour change. Such approaches to intervention refinement have been adopted before in asthma, weight management, alcohol and smoking research [29–31].

## Conclusions

The partnership between UCL and CLIC Sargent allowed a process of intervention development to be followed which recognizes and listens to the specific needs of TYACS. Data on TYACS current health behaviours and preferences regarding health behaviour change intervention delivery were gathered and combined with TYA health professionals perspectives. This data was used to inform the development of a collection of health behaviour change intervention materials. The preliminary evaluation study demonstrates the utility of these health behaviour change resources. It confirmed that TYACS are interested in receiving health behaviour information and are receptive to the idea of engaging with behaviour change interventions. Moreover, the evaluation study allowed the identification of several features of the information which could be improved upon and highlighted some key issues which should be considered in the further development and implementation of the resources. The next step is to carry out feasibility and pilot randomized controlled trials exploring uptake and effect of the resources on behaviour change among young people with cancer.

## Additional file

Additional file 1: Reviewers report. (DOCX 17 kb)

## Abbreviations

TYACS: Teenage and young Adult cancer survivor
