# Supplementary material for: The development and user evaluation of health behaviour change resources for teenage and young adult Cancer survivors
Source: Res Involv Engagem. 2019 Feb 15;5:9. doi: 10.1186/s40900-019-0142-6 (PMC6377718; doi:10.1186/s40900-019-0142-6)
Supplement: Supplementary file 1 — Reviewers report. (DOCX 17 kb) [file 40900_2019_142_MOESM1_ESM.docx]

**Energy Balance & Cancer Research Group**

**Department of Behavioural Science & Health**

**University College London**

Dear Editors in Chief,

*RE: The Development and User Evaluation of Health Behaviour Change Resources for Teenage and Young Adult Cancer Survivors*

Thank you for your prompt response to our submission to Research Involvement and Engagement. We would like to thank you for the opportunity to revise the manuscript and for the reviewers for their constructive comments. We have now submitted an updated manuscript for your consideration.

We have carefully considered the reviewers comments and addressed each in turn below. All changes within the manuscript are highlighted in yellow.

Best Wishes,

Gemma Pugh

**Reviewer reports:
Reviewer #1: 1. It is not clear the actual aim of this paper. It appears the authors highlighted the main aim of the entire programme.**

*As stated within the abstract the purpose of this paper was to summarise key findings from the development work, the resulting lifestyle intervention, and new data from a preliminary evaluation study exploring TYACS and TYA health professionals’ views on the relevance, appeal and usability of the intervention resources. We now provide specific reference to this within the main body of the manuscript. Thank you for suggesting this important clarification.*

*Page 4. ‘The aim of this programme of work was to develop a health behaviour intervention specifically for TYACS. In this paper we summarise key findings from the development work, the resulting lifestyle intervention, and new data from a preliminary evaluation study exploring TYACS and TYA health professionals’ views on the relevance, appeal and usability of the intervention resources. Specifically within the preliminary evaluation study we aimed to determine i) if the health behaviour information developed as part of the proposed intervention meets the needs of TYACS and TYA health professionals and ii) if TYACS and TYA health professionals are receptive to the inclusion of discrete behaviour change techniques within the resources.’*

**2. Line 49, authors stated that several frameworks have been published on interventions in a logical evidence based way. However, only one reference was provided. Can authors provide additional references to aid readers appreciate the other published interventions.**

*The reference cited refers to a review of intervention development studies and updates in the field of intervention development. We also cite the ‘The Behaviour Change Wheel: A Guide to Designing Interventions’ published by Michie and colleagues. Both references discuss the wide range of intervention development frameworks in existence and the application of these in various fields of research.*

*Ref 13. Hoddinott, P., A new era for intervention development studies. Pilot and Feasibility Studies, 2015.* ***1****(1): p. 36.*

*Ref 14.	Michie, S., L. Atkins, and R. West, The Behaviour Change Wheel: A Guide to Designing Interventions 2014.*

**3. In terms of showing the results, I suggest that the authors should include more background information of the participants i.e. TYACS years of living with the conditions as well as the health professional's years of experience. This is if these information is available…..**

*We have now included more detail on the demographics of participants. Thank you for this suggestion.*

*Page 7 ‘A total of 18 TYACS (mean age: 20±3.12 years, 65% female) and 19 TYA health professionals (62% nurses, 19% social workers, and 5% physicians) took part in the online survey. Most TYACS had completed active treatment (83.4%) and reported a diagnosis of either leukaemia or lymphoma (55.5%). TYA health professionals predominantly worked in principal treatment centres with TYA patients with varying types of cancer. Most professionals (81%) had worked with TYA cancer patients for more than 2 years.’*

**Reviewer #2: Interesting article around a subject that appears to be in it's infancy despite the need identified. However, I think that more engaging methods to get the views of the TYACS could have been employed. The author mentioned that there was a drop out rate because of survey fatigue and this is not surprising. This reduced the number of an already small number of respondents.**

*We thank reviewer 2 for highlighting the relevance of the article. We agree a more engaging method may have resulted in a greater participant response. We used the online survey as we wanted to keep participant burden minimal, we recognise that in the future workshops reviewing the content may result in better feedback.*

**Reviewer #3: This is a very interesting and informative article detailing the intensive exploration into teenage and young adults' views and ideas around behaviour change interventions and resources designed for their specific cancer patient community.

Patients and professionals equally contributed to the project ,a collaboration equally between academic researchers and the CLIC Sergeant charity.

One insight provided is that some young participants experienced fatigue from the surveys ,leading to high drop-out rates after the first round of tests .This issue is noted and acknowledged with future strategies indicated to look to look at this area of need. Given that individuals each have different learning styles and requirements, the potential in this study for further development of innovative and effective ways and tools to support and encourage positive health attitude and behaviour outcomes in this particular patient group.

Future research plans evinces are realistic and realisable.**

*We thank reviewer 3 for highlighting the range of work we carried out to understand teenage and young adults' views and ideas around behaviour change interventions and resources. Our future work will look to developing innovative methods to support and encourage positive health attitude and behaviour outcomes in this patient group.*


**Reviewer #4: It would appear that the authors have responded to comments from the previous reviewers.**

**However, I have the following minor additional comments:
1) It is not clear as to whether or not the teenage and young adult cancer survivors (TYACS) were paid for their time and the rationale for this decision.**

*Participants did not receive an incentive for taking part in the evaluation study described within the manuscript. We did provide a prize draw incentive within the study exploring TYACS information needs and preferences (Pugh et al., 2018. JAYAO) and provided a shopping voucher incentive within the qualitative study exploring young peoples past experience of receiving lifestyle advice (Pugh et al., 2017. Cancer Nurs). Health professionals did not receive any incentive for their participation within the previous research study (Pugh et al., 2017. Support Care Cancer) or the current study.*

2) Did the researchers consider multimedia formats such as films as part of the resources assembled for the intervention.

*We did not include multimedia formats such as films or videos within the resources as within our previous research (Pugh et al., 2018 JAYAO) less than 10% of young people said they watched videos on YouTube to get information about physical activity, diet, smoking or alcohol consumption.*
